# Plasma Interferon-Gamma-Inducible Protein-10 Levels Are Associated with Early, but Not Sustained Virological Response during Treatment of Acute or Early Chronic HCV Infection

**DOI:** 10.1371/journal.pone.0080003

**Published:** 2013-11-20

**Authors:** Jordan J. Feld, Jason Grebely, Gail V. Matthews, Tanya Applegate, Margaret Hellard, Alana Sherker, Vera Cherepanov, Kathy Petoumenos, Barbara Yeung, John M. Kaldor, Andrew R. Lloyd, Gregory J. Dore

**Affiliations:** 1 Toronto Centre for Liver Disease, McLaughlin-Rotman Centre for Global Health, University of Toronto, Toronto, Canada; 2 The Kirby Institute for Infection and Immunity in Society, University of New South Wales, Sydney, Australia; 3 HIV/Immunology/Infectious Diseases Clinical Services Unit, St Vincent’s Hospital, Sydney, Australia; 4 Burnet Institute, Melbourne, Australia; 5 Infectious Diseases Unit, The Alfred Hospital, Melbourne, Australia; 6 Inflammation and Infection Research Centre, School of Medical Sciences, University of New South Wales, Sydney, Australia; University of Sydney, Australia

## Abstract

**Background:**

High plasma levels of interferon-gamma inducible protein-10 (IP-10) have been shown to be associated with impaired treatment response in chronic hepatitis C virus (HCV) infection. Whether IP-10 levels predict treatment in acute HCV infection is unknown.

**Methods:**

Patients with acute or early chronic HCV infection from the Australian Trial in Acute Hepatitis C (ATAHC) cohort were evaluated. Baseline and on-treatment plasma IP-10 levels were measured by ELISA. IL28B genotype was determined by sequencing.

**Results:**

Overall, 74 HCV mono-infected and 35 HIV/HCV co-infected patients were treated in ATAHC, of whom 89 were adherent to therapy and were included for analysis. IP-10 levels correlated with HCV RNA levels at baseline (r = 0.48, *P*<0.001) and during treatment. Baseline IP-10 levels were higher in patients who failed to achieve rapid virological response (RVR). Only one patient with a plasma IP-10 level >600 pg/mL achieved RVR. There was no association with IP-10 levels and early virological response (EVR) or sustained virological response (SVR).

**Conclusions:**

Baseline IP-10 levels are associated with early viral kinetics but not ultimate treatment outcome in acute HCV infection. Given previous data showing that patients with high baseline IP-10 are unlikely to spontaneously clear acute HCV infection, they should be prioritized for early antiviral therapy.

## Introduction

The development of direct-acting antivirals (DAAs) has markedly improved response rates for chronic hepatitis C virus (HCV) infection. However, treatment during the acute phase of HCV infection is associated with excellent treatment outcomes with interferon-based therapy without DAAs [1]. In the initial study of interferon treatment for acute HCV, Jaeckel and colleagues found a striking 98% rate of SVR [2]. Many subsequent studies have shown similarly good outcomes with peginterferon therapy with or without ribavirin in acute HCV infection [3]. The reasons for the marked difference in response rates between acute and chronic infection remain unclear.

Great efforts have gone into understanding the reasons for treatment failure in chronic HCV infection. Virological and host factors have been identified as predictors of treatment response, however the underlying mechanisms explaining the associations are poorly understood. Evaluation of hepatic gene expression profiles has shown that heightened expression of interferon-stimulated genes (ISG) in the liver prior to receiving interferon therapy is associated with treatment failure [4], [5]. Future non-responders to therapy have near-maximal ISG expression at baseline with little or no further gene induction in response to interferon treatment [5], [6]. The reason for ISG pre-activation is not known but recent identification of single nucleotide polymorphisms (SNPs) near the IL28B gene that are associated with spontaneous and treatment-induced clearance of HCV may be very important [7]–[9]. Patients with the unfavorable IL28B genotype have higher levels of hepatic ISG expression, suggesting that ISG pre-activation may be the phenotypic expression of the IL28B genotype [10], [11]. However, although the recent recognition of a novel IFN-lambda, designated IFN-lambda 4, which is upstream of IL28B and may underlie the association of the IL28B genotype with treatment response, the mechanisms by which it affects gene expression patterns and ultimately treatment response remain unclear [12].

Evaluation of hepatic gene expression is costly and impractical, greatly limiting its clinical utility. However, serum markers may offer similar prognostic information. Multiple studies have shown that levels of the ISG, interferon-gamma inducible protein-10 (IP-10, CXCL10), are associated with treatment outcome in chronic HCV infection [13]–[15]. Patients with high IP-10 at baseline are much less likely to achieve SVR. Lagging et al found that only 21% of patients with baseline IP-10 levels above 600 pg/mL achieved SVR compared to 68% of those with IP-10 values below 150 pg/mL [14]. IP-10 is an interferon-inducible chemokine that binds the CXR3 on lymphoid cells driving them to home to sites of inflammation [16]. In chronic HCV infection, serum IP-10 levels correlate with IP-10 mRNA levels in hepatocytes suggesting that serum IP-10 level may be a useful surrogate for overall ISG activation in the liver [17].

The marked differences in treatment outcome between acute and chronic HCV infection imply fundamental changes occur upon evolution to chronic infection that limit future interferon responses in some patients. The relative lack of liver biopsy material in acute infection has limited evaluation of hepatic gene expression and its correlation with outcome. However, if IP-10 serves as a serum surrogate of ISG expression in the liver, determination of IP-10 concentrations in acute infection may shed light on the degree of ISG activation in acute infection. It may also be a useful clinical tool to predict treatment outcome. To assess the role of IP-10 during treatment of acute HCV infection, plasma levels of IP-10 were measured in patients treated in the Australian Trial in Acute Hepatitis C (ATAHC) cohort.

## Methods

### Patients

ATAHC is a multicentre, prospective cohort study of the natural history and treatment of acute and early chronic HCV infection [18]. For inclusion, patients had to have acute or early chronic HCV infection defined by an initial positive anti-HCV antibody test within 6 months of enrolment and either 1) a negative anti-HCV antibody test within 2 years prior to the initial positive anti-HCV antibody test or 2) acute clinical hepatitis within 12 months of the initial positive anti-HCV antibody result. The protocol was reviewed and approved by the research ethics board of St. Vincent’s Hospital, Sydney Human Research Ethics Committee and the University Health Network and all patients provided informed written consent.

### Treatment

Patients who were HCV RNA positive at enrolment in the ATAHC cohort were offered antiviral therapy. HCV mono-infected patients were offered peginterferon-α 2a 180 µg per week for 24 weeks irrespective of viral genotype. After the first two patients with HIV-HCV co-infection failed to respond to peginterferon monotherapy, the protocol was amended to treat co-infected patients with peginterferon-α 2a and ribavirin (800 mg daily for G2/3 and 1000–1200 mg for genotype 1). HCV RNA levels were assessed at screening and again at treatment weeks 0, 4, 8, 12 and 24 with follow-up samples at weeks 36, 48 and every 12 weeks through week 144. Additional plasma samples were collected with each HCV RNA measurement and stored at −80°C. Standard treatment definitions were used. A rapid virological response (RVR) indicated HCV RNA undetectability (<10 IU/ml) at treatment week 4. Clearance of viremia by week 12 was deemed a complete early virological response (cEVR) and by week 24, an end-of-treatment response (ETR). Patients were deemed to have achieved a sustained virological response (SVR) if HCV RNA remained undetectable 24 weeks after the completion of therapy. Among individuals with an ETR who were subsequently HCV RNA positive, HCV RNA sequencing was performed and compared to baseline to distinguish viral relapse (similar HCV RNA sequences) from reinfection (different HCV RNA sequences) [19].

### Laboratory Tests

HCV RNA was performed with a qualitative assay (TMA assay, Versant, Bayer, Australia) with a lower limit of detection of 10 IU/mL and if positive, was repeated with a quantitative assay (Versant HCV RNA 3.0 Bayer, Australia) with a lower limit of detection of 615 IU/mL. HCV RNA levels that were detectable but non-quantifiable were set to 310 IU/mL (the midpoint between the qualitative and quantitative assay). Plasma IP-10 was measured from baseline (week 0) and follow-up samples using an in-house enzyme-linked immunosorbant assay (ELISA) standardized to a commercial ELISA (Bio-Rad, Hercules, CA) [20]. IL28B genotype was determined by direct sequencing of the rs8099917 and rs12979860 SNPs, as described previously [21], [22].

### Statistical Analysis

The association between baseline plasma IP-10 and treatment response was evaluated. Analyses were restricted to individuals who were 80% adherent to peginterferon therapy, defined as receipt of ≥80% of scheduled peginterferon doses for ≥80% of the scheduled treatment period. For participants who terminated at 12 weeks due to virological non-response, the scheduled treatment period was defined as 12 weeks. Median IP-10 levels were compared between those who did and did not achieve RVR, cEVR, ETR and SVR using the non-parametric Mann-Whitney U test. Based on data from chronic HCV infection [14], [15], [23], [24], the association between treatment response and IP-10 levels above or below 150 and 600 pg/mL thresholds or as a continuous variable was evaluated using logistic regression analysis. Other factors included in regression models with treatment outcome or IP-10 level were age, sex, IL28B genotype, viral genotype, HCV RNA titre, estimated duration of infection, symptomatic presentation and HIV co-infection. Factors significant (p<0.10) by univariate analysis and factors with potential for significant confounding were included in forward and backward stepwise multivariable models. Normally distributed continuous data were compared using the Students t-tests and non-normally distributed parameters were either log10-transformed or compared using the Mann-Whitney U test. Pearson or Spearman’s correlation coefficients were used to determine associations between parametric variables and non-parametric variables respectively. P-values <0.05 were considered significant. All analyses were performed using Stata 12.1 (College Station, TX).

## Results

### Treatment Results

As previously described, 74 HCV mono-infected and 35 HIV/HCV co-infected patients were treated in the ATAHC cohort [18]. The baseline characteristics of the patients are shown in Table 1. To avoid spurious conclusions, analysis was limited to those who were compliant with treatment (≥80% of the prescribed peginterferon regimen) (n = 89). Among HCV mono-infected adherent patients, 55% achieved RVR, 76% cEVR, 82% ETR and 72% SVR. In those with HIV/HCV co-infection, 41% achieved RVR, 85% cEVR, 81% ETR and 75% SVR.

**Table 1:** Baseline characteristics among treated HCV and HCV/HIV infected participants with recently acquired HCV infection and adherent to PEG-IFN therapy (n = 89).

	Overall(n = 89) n (%)	HCV infected (PEG-IFN) (n = 57) n (%)	HCV/HIV infected (PEG-IFN/ribavirin) (n = 32) n (%)
Male Sex	67 (75)	35 (61)	32 (100)
Mean age, yrs (SD)	34.8 (10.5)	31.0 (9.2)	41.6 (9.4)
Tertiary education or greater	47 (53)	24 (42)	23 (72)
Full-time or part-time employment	43 (48)	21(37)	22 (69)
Injecting drug use ever	66 (74)	48 (84)	18 (56)
Injection drug use in previous 30 days	26 (30)	22 (39)	4 (13)
Social support			
≤14	52 (58)	27 (47)	25 (78)
≥14	28 (31)	23 (40)	5 (16)
Missing	9 (10)	7 (12)	2 (6)
IL28B genotype			
rs8099917			
GG	4 (5)	1 (2)	3 (10)
GT	31 (36)	23 (42)	8 (26)
TT	51 (59)	31 (56)	20 (65)
rs12979860			
CC	42 (48)	24 (43)	18 (58)
CT	36 (41)	27 (48)	9 (29)
TT	9 (10)	5 (9)	4 (13)
Mode of HCV acquisition			
Injecting drug use	60 (67)	48 (84)	12 (38)
Sexual	23 (26)	4 (7)	19 (59)
Other	6 (7)	5 (9)	1 (3)
Estimated duration of HCV infection ≥26 wks*	67 (75)	46 (81)	21(66)
Presentation of recent HCV ¥			
Acute clinical (symptomatic)	34 (38)	20 (35)	14 (44)
Acute clinical (ALT >400 IU/mL)	21 (24)	11 (19)	10 (31)
Asymptomatic seroconversion	34 (38)	26 (46)	8 (25)
HCV RNA ≥400,000 IU/mL*	36 (40)	18 (32)	18 (56)
HCV genotype			
Genotype 1	50 (56)	33 (58)	17 (53)
Genotype 2	5 (6)	1 (2)	4 (13)
Genotype 3	33 (37)	22 (39)	11 (34)
Mixed HCV genotype 1/3	1 (1)	1 (2)	0 (0)

* at baseline,

¥ denominator is total number of people reporting documented illness.

SD, standard deviation.

### Plasma IP-10 Levels by Baseline Characteristics

**Figure 1:**
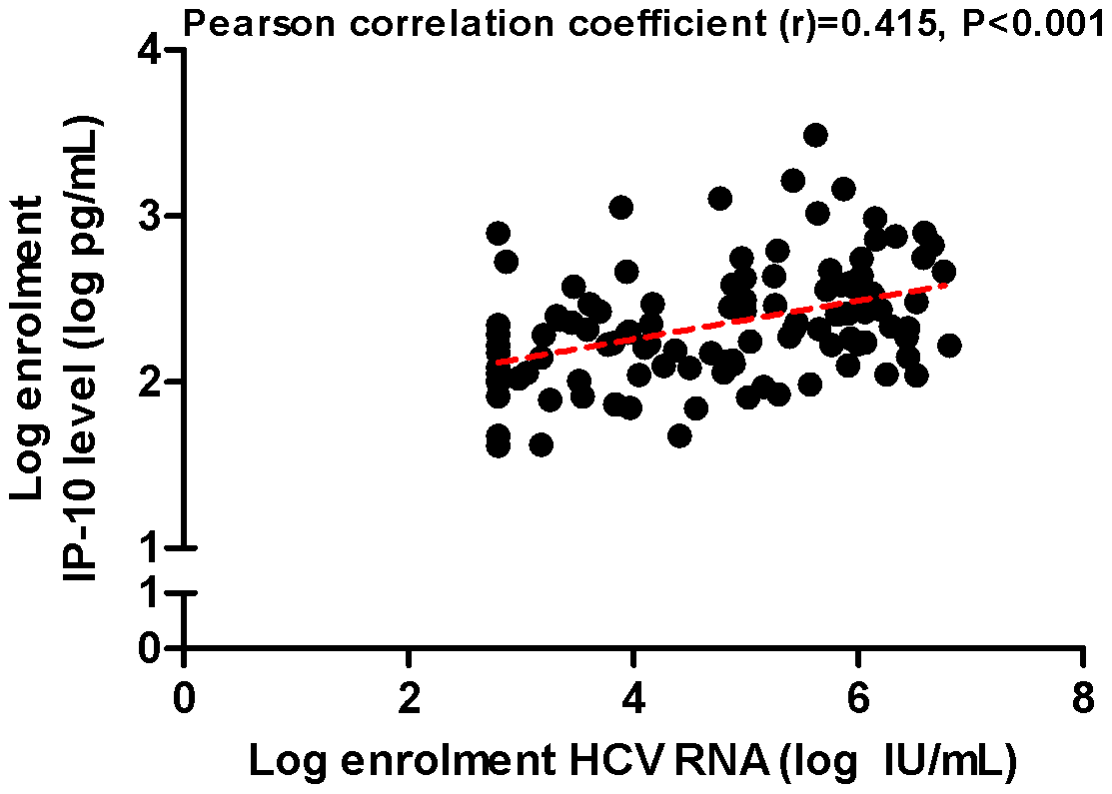
Correlation of baseline plasma IP-10 and HCV RNA titre. The correlation between baseline plasma IP-10 level and serum HCV RNA titre was evaluated using Pearson’s correlation co-efficient.

**Figure 2:**
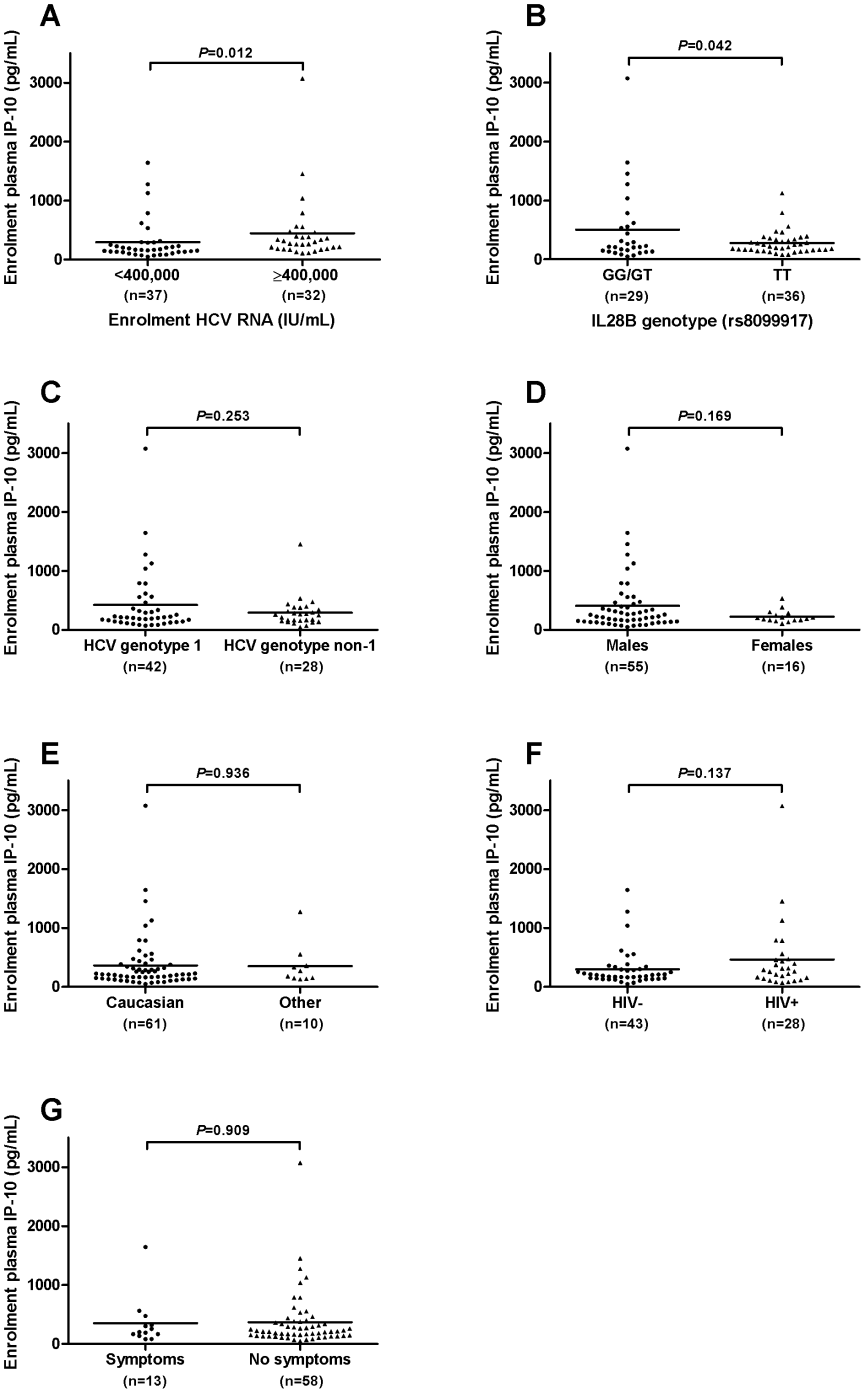
Baseline plasma IP-10 values according to patient characteristics. Baseline plasma IP-10 levels are shown by: a) HCV RNA above or below 400,000 IU/mL b) IL28B genotype c) HCV genotype (1 vs. non-1) d) sex e) ethnicity (Caucasian vs. other) f) HIV status and g) the presence or absence of symptoms at presentation. Mean plasma IP-10 levels were compared using the student’s t-test with Welch’s correction for unequal variances as appropriate.

Plasma IP-10 levels at baseline ranged from 47 to 3,071 pg/mL and were positively skewed with a median of 212 pg/mL and a mean of 365±456 pg/mL. IP-10 levels correlated with HCV RNA titre (p<0.0001, r = 0.42) (Figure 1) and mean baseline IP-10 levels were higher in those with HCV RNA above 400,000 IU/mL (522±110 vs. 246±42 pg/mL, *P* = 0.012) (Figure 2a). Mean IP-10 levels were higher in patients with the treatment unfavorable IL28B genotype at rs8099917 (non-TT: 505±122 vs. TT: 278±31, *P* = 0.04). Mean IP-10 levels at baseline did not differ by HCV genotype (genotype 1∶420±85 pg/mL vs. genotype non-1∶291±49 pg/mL, *P* = 0.25), sex (men: 406±67 pg/mL vs. women: 227±27 pg/mL, *P* = 0.17), ethnicity (Caucasian: 367±61 pg/mL vs. Non-Caucasian: 355±110 pg/mL, p = 0.94), HIV status (HIV +ve: 465±114 vs. HIV –ve: 300±48, *P* = 0.14) or the presence of symptoms at presentation (symptomatic: 352±114 vs. asymptomatic: 368±61, *P* = 0.91) (Figure 2 b–g).

### Plasma IP-10 Levels and Treatment Response

**Figure 3:**
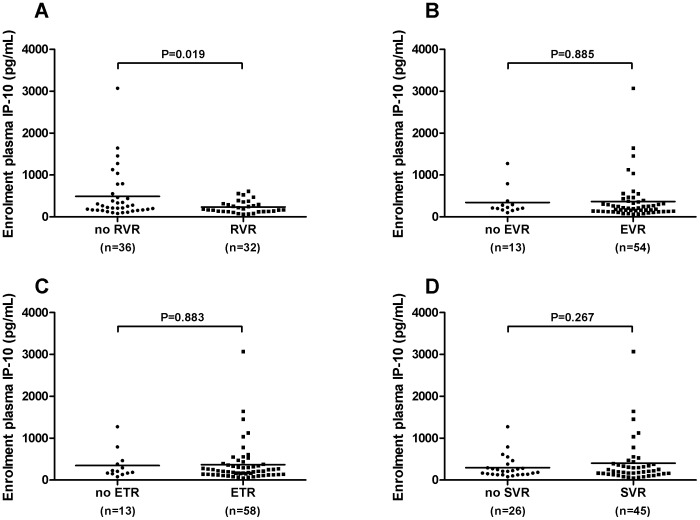
Baseline plasma IP-10 according to virological responses. Baseline IP-10 values are shown in patients who did and did not achieve: a) RVR b) EVR c) ETR and d) SVR. Results are shown for the entire treated cohort. IP-10 levels were compared using the student’s t-test with Welch’s correction for unequal variances as appropriate. RVR rapid virological response; EVR early virological response; ETR end of treatment response; SVR sustained virological response.

**Figure 4:**
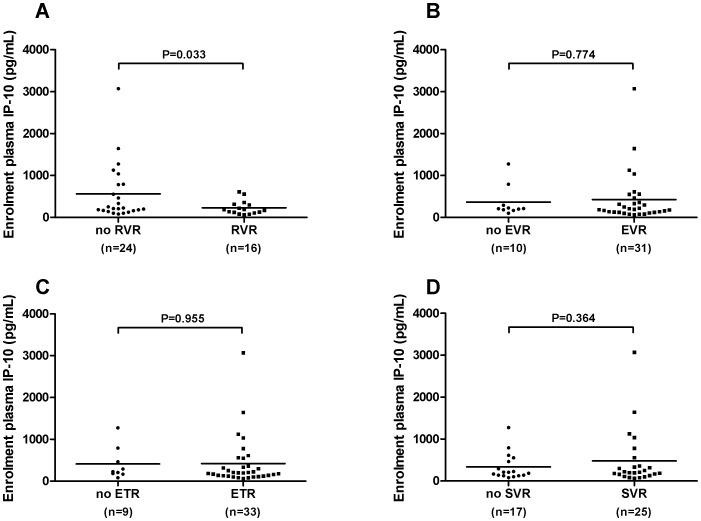
Baseline plasma IP-10 according to virological responses in genotype 1 patients only. Baseline IP-10 values are shown in genotype 1 patients who did and did not achieve: a) RVR b) EVR c) ETR and d) SVR. IP-10 levels were compared using the student’s t-test with Welch’s correction for unequal variances as appropriate. RVR rapid virological response; EVR early virological response; ETR end of treatment response; SVR sustained virological response.

**Figure 5:**
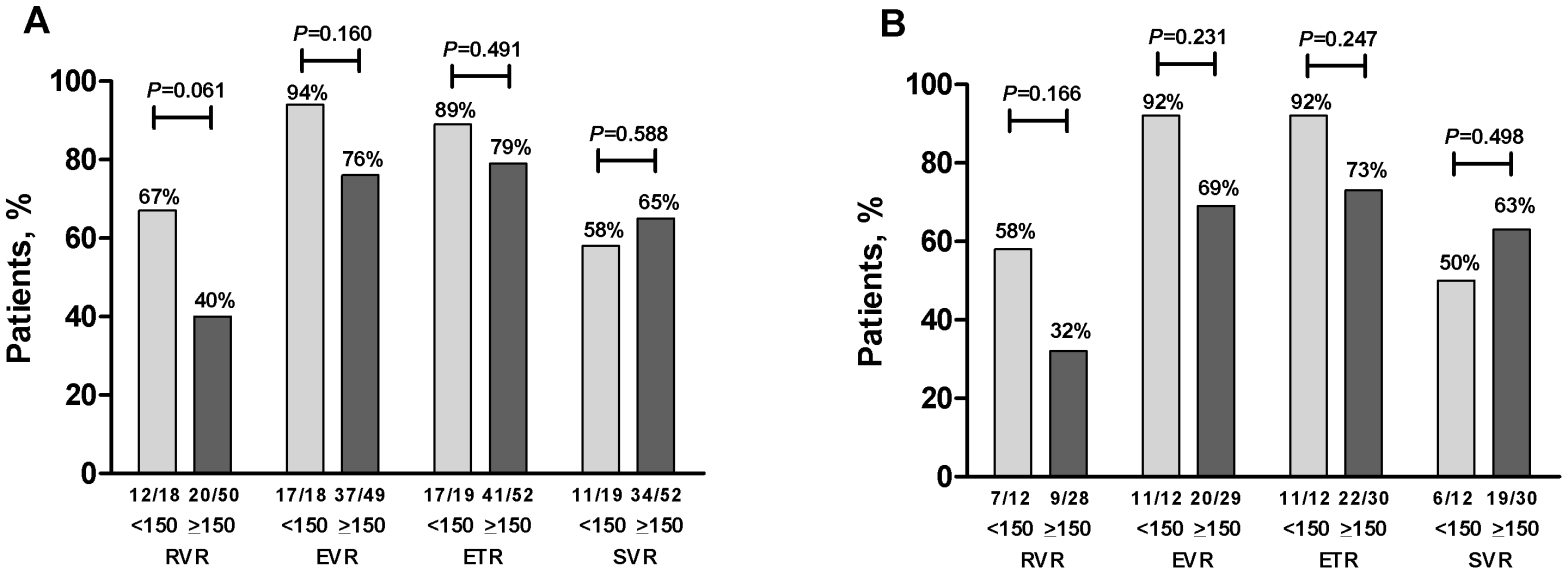
Response based on baseline IP-10 level above or below 150 pg/mL among adherent individuals. The proportion of patients with baseline IP-10 levels above or below 150 pg/mL who achieved RVR, cEVR, ETR and SVR are shown for a) the whole cohort and b) patients with genotype 1 infection. Responses were compared using Fisher’s Exact Test.

Mean IP-10 levels at baseline were lower in patients who achieved RVR than in those who did not (RVR: 240±26 pg/mL vs 492±100 pg/mL, *P* = 0.024) (Figure 3a) with similar results seen restricting the analysis to those with genotype 1 infection (RVR: 230±41 pg/mL vs 560±140 pg/mL, *P* = 0.033) (Figure 4a). Only 1 of 10 (10%) patients with baseline IP10 level above 600 pg/mL (616 pg/mL) had an RVR compared to 30 of 58 (52%) of those with baseline IP-10 below this threshold (*P* = 0.017). Conversely, patients with low baseline IP-10 (<150 pg/mL) were more likely to achieve RVR (IP-10<150 pg/mL: 12 of 18 (67%) RVR vs IP-10≥150 pg/mL: 20 of 50 (40%) RVR, *P* = 0.061), with similar results with the whole cohort and in those with genotype 1 infection (Figure 5a, b). IP-10 levels did not correlate with estimated duration of infection (r = -0.06, *P* = 0.60) and the relationship between RVR and IP-10 remained significant after controlling for estimated duration of infection (adjusted OR for RVR with IP-10<150 pg/mL: 0.29, 95% CI 0.09–0.94, *P* = 0.039).

Baseline IP-10 levels did not differ between patients who did and did not achieve cEVR, ETR or SVR in the whole cohort (Figure 3b–d) or in patients with genotype 1 infection only (Figure 4b–d). Furthermore, the proportion of patients with cEVR, ETR and SVR did not differ between those with baseline IP-10 above or below 150 pg/mL, 600 pg/mL or any other threshold value. Notably, although only one of the 10 adherent patients with baseline IP-10 levels >600 pg/mL achieved RVR, 6 of these 10 patients went on to SVR.

### Association of Plasma IP-10 Levels with IL28B Genotype and HCV RNA

Notably, IL28B genotype was not associated with RVR (TT vs non-TT: OR 1.67, 95% CI 0.68–4.1, *P* = 0.26) and did not confound the relationship between IP-10 levels below 150 pg/mL and RVR (adjusted OR 0.28, 95% CI 0.08–0.96, *P* = 0.043). However, the baseline HCV RNA level was strongly associated with early treatment outcome. Only 7 of 33 (21%) patients with HCV RNA levels above 400,000 IU/mL achieved RVR including 0 of 7 who also had an IP-10 level >600 pg/mL, compared to 36 of 51 (71%) with RNA titres below 400,000 IU/mL (*P*<0.001). After controlling for HCV RNA level, IP-10 below 150 pg/mL was no longer associated with RVR (OR 0.66, 95% CI 0.19–2.35, *P* = 0.53). By multivariate logistic regression, HCV RNA below 400,000 IU/mL, genotype non-1 and an estimated duration of infection less than 6 months remained significantly associated with RVR (Table 2).

**Table 2:** Predictors of rapid virological response among treated HCV and HCV/HIV infected participants with recently acquired HCV infection adherent to therapy (n = 85*).

	RVR(n = 43)n, (%)£	Unadjustedodds ratio(95% CI)	*P*	*P*overall	Adjustedodds ratio(95% CI)	*P*
Male Sex (vs. female sex)	31 (49)	1.24 (0.47, 3.28)	0.667	–	–	–
Injection drug use in previous 30 days (vs. none)	13 (50)	1.00 (0.40, 2.52)	1.000	–	–	–
Social support						
≤14	23 (46)	1.00	–	–	–	–
>14	17 (65)	2.22 (0.83, 5.91)	0.112	0.161	–	–
Missing	3 (33)	0.59 (0.13, 2.61)	0.484	–	–	–
IL28B genotype						
rs8099917 favorable TT (vs. GT/GG)	27 (55)	1.67 (0.68, 4.06)	0.262	–	–	–
rs12979860 favorable CC (vs. CT/TT)	22 (55)	1.54 (0.65, 3.67)	0.326	–	–	–
Estimated duration of HCV infection of ≥26 wks atbaseline (vs. <26 weeks)	30 (46)	0.46 (0.16, 1.31)	0.145	–	0.23 (0.06, 0.87)	0.031
Presentation of recent HCV						
Acute clinical (symptomatic)	18 (56)	1.00	–	–	–	–
Acute clinical (ALT >400 IU/mL)	7 (35)	0.42 (0.13, 1.33)	0.139	0.288	–	–
Asymptomatic seroconversion	18 (55)	0.93 (0.35, 2.48)	0.890	–	–	–
HCV RNA ≥5.6 log_10_ (IU/mL) at baseline(vs. <5.6 log_10_)	7 (21)	0.11 (0.04, 0.30)	<0.001	–	0.09 (0.03, 0.30)	<0.001
HCV genotype						
Genotype 1¥	18 (39)	1.00	–	–	–	–
Genotype 2/3	22 (61)	2.44 (1.00, 5.98)	0.050	–	3.31 (1.11, 9.90)	0.032
Received ribavirin/HIV infection	13 (43)	0.64 (0.26, 1.56)	0.324	–	0.71 (0.23, 2.25)	0.563
IP-10 at enrolment					Adjusted	
Log IP-10/per unit increase	–	0.17 (0.03, 0.84)	0.030	–	0.30 (0.04, 2.19)	0.235
≥150 pg/mL	20 (40)	0.33 (0.11, 1.03)	0.057	–	0.44 (0.11, 1.80)	0.252
≥380 pg/mL	5 (29)	0.37 (0.11, 1.20)	0.100	–	0.44 (0.11, 1.80)	0.251
≥600 pg/mL	1 (11)	0.11 (0.01, 0.96)	0.046	–	0.19 (0.02, 2.25)	0.189

* four of the 89 adherent participants did not have evaluable HCV RNA at week 4 and were excluded from this analysis,

¥ includes one participant with mixed genotype 1/3 infection,

£ row percentage (e.g. proportion with cRVR). RVR, rapid virological response; CI, confidence interval.

**Figure 6:**
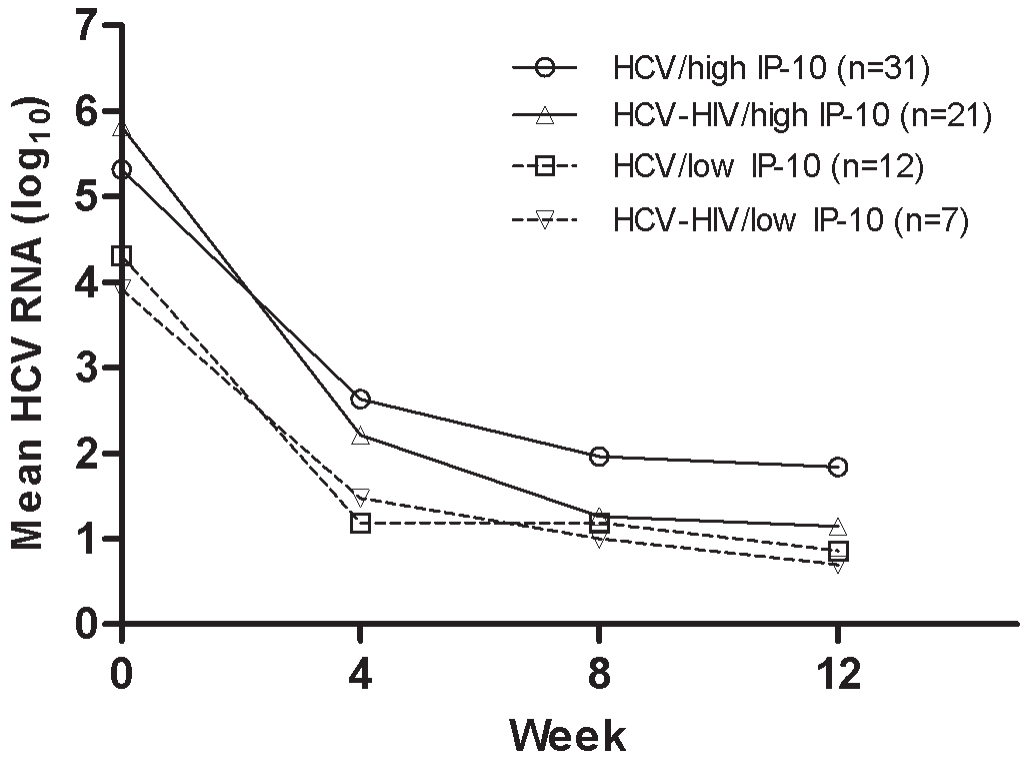
Early virological decline among adherent participants with recent HCV. The decline in HCV RNA during the first 12 weeks of treatment is shown in patients with high baseline plasma IP-10 in those with HCV monoinfection (PEG-IFN) (>150 pg/mL, circles and solid line) or HIV/HCV co-infection (PEG-IFN/ribavirin) (≥150 pg/mL, upward triangles and solid line). The HCV RNA decline is also shown for patients with low baseline plasma IP-10 levels and HCV monoinfection (<150 pg/mL, downward triangles and dotted line) or HCV/HIV co-infection (<150 pg/mL, squares and dotted line).

IP-10 levels declined with the fall in HCV RNA. There was a correlation between the change in log IP-10 and change in log HCV RNA from baseline to week 4 (r = 0.52, *P* = 0.06) and baseline to week 12 (r = 0.31, *P* = 0.015). Beyond week 4, the decline in HCV RNA was more rapid in patients who received ribavirin, but only in those who started with a baseline IP-10>150 pg/mL. In those with low baseline IP-10, the decline was similar with and without ribavirin therapy (Figure 6). IP-10 declined in patients who achieved an SVR but returned to near baseline levels in those who failed to reach this endpoint (Delta log IP-10 baseline to week 48 in SVR: 0.42±0.06 vs. No SVR: 0.05±0.07, *P* = 0.001).

## Discussion

Baseline levels of IP-10 have been shown to correlate with treatment outcome in chronic HCV infection, with higher levels predicting treatment failure. In this cohort of acute and early chronic HCV infection, baseline IP-10 levels were predictive of early viral kinetics but not ultimate treatment outcome. These findings may have important clinical and pathophysiological significance.

Patients with low baseline IP-10 levels (<150 pg/mL) were more likely to achieve an RVR than those with higher IP-10 levels, even after controlling for estimated duration of infection and IL28B genotype. Beyond this early time-point, baseline IP-10 level and HCV RNA titre were no longer predictive of treatment response.

**Figure 7:**
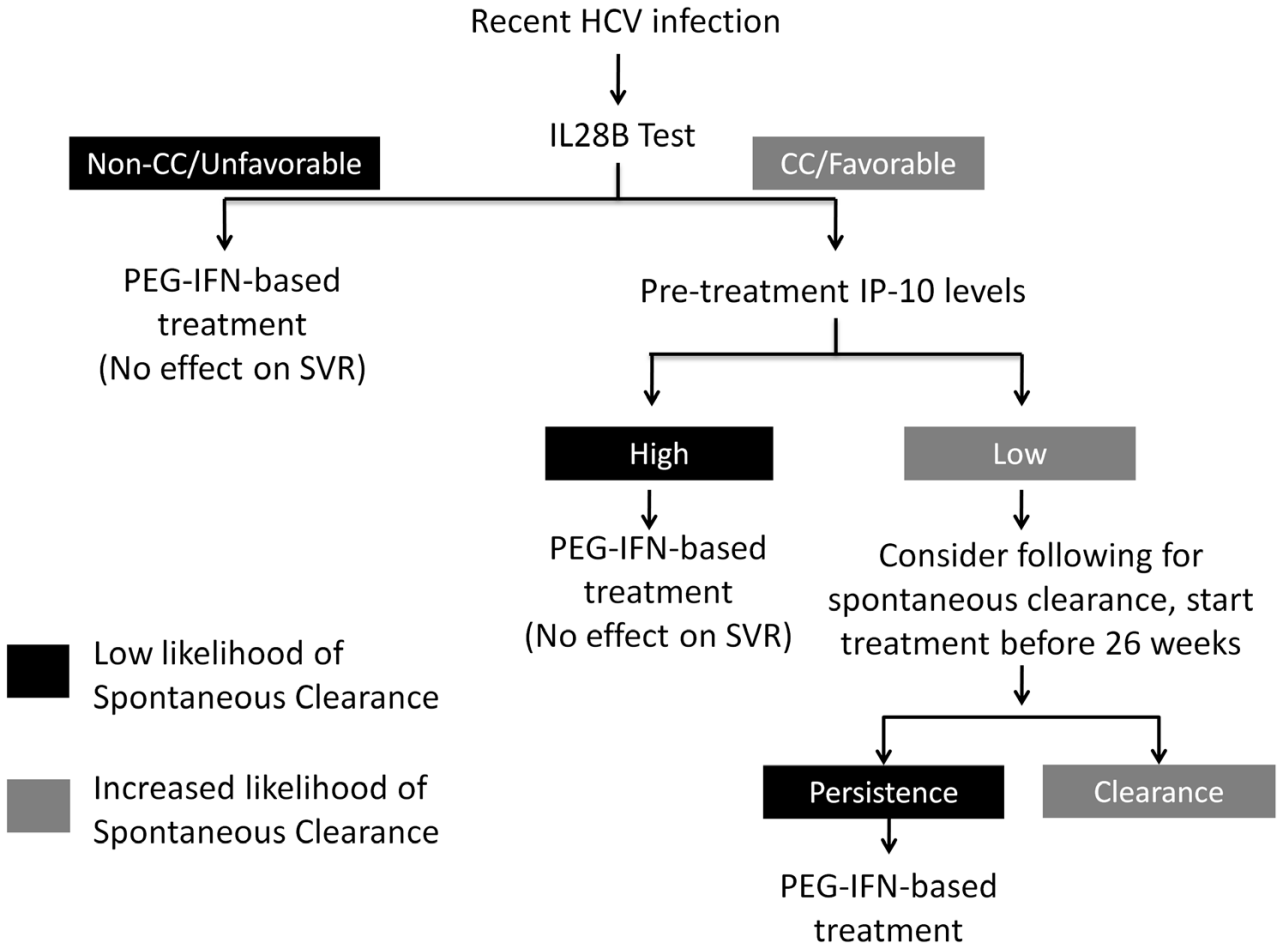
Proposed algorithm for incorporation of plasma IP-10 testing into clinical management of acute HCV infection.

The finding that IP-10 levels were not predictive of ultimate treatment outcome may have important clinical implications. We recently reported that higher IP-10 levels were associated with failure of spontaneous HCV clearance in acute infection [21]. No patients with a baseline IP-10 level above 380 pg/mL went on to clear virus without therapy. Furthermore high IP-10 during acute infection may be predictive of persistently high IP-10 after progressing to chronicity [25], which may impair future treatment responses. High pre-treatment IP-10 levels are associated with reduced rates of SVR during PEG-IFN/RBV treatment of chronic HCV [14], [15], [17], [23], [26], [27]. An unfavorable IL28B genotype and absence of symptoms are also associated with failure of spontaneous clearance, but like IP-10 [22], [28], they do not appear to affect the response to treatment during acute HCV. Collectively these data suggest that patients with high baseline IP-10 levels (>150 pg/mL), particularly those with asymptomatic disease and/or an unfavourable IL28B genotype, should be prioritized for early antiviral therapy because they are unlikely to achieve spontaneous clearance but still have a high likelihood of SVR with treatment during the acute phase of infection (Figure 7).

The correlation of IP-10 levels with early but not late treatment responses is interesting and may offer some clues to explain the markedly different outcomes of interferon-based treatment in acute and chronic HCV infection. In chronic infection future non-responders to treatment have strong activation of ISGs in the liver prior to receiving interferon therapy [4], [5]. With near maximal ISG expression at baseline, ISGs cannot be further induced in non-responders despite interferon-based therapy [5], [6]. IP-10 in the serum appears to be a reflection of ISG preactivation in the liver; IP-10 serum levels correlate with IP-10 mRNA levels in liver biopsy tissue [17] and high serum IP-10 levels are associated with treatment non-response [14]. In this study of acute and early chronic HCV infection, patients with high baseline IP-10 responded less well to interferon with lower rates of RVR. However, unlike in the setting of established chronic infection, even patients with high baseline IP-10 levels with a poor initial response to therapy, were ultimately able to suppress virus and achieve SVR. This is particularly noteworthy given that apart from the HIV/HCV coinfected group, all ATAHC patients received PEG-IFN monotherapy. The difference in outcome in these scenarios is likely key to the difference in treatment response in acute and chronic HCV.

There may be differences in both innate and adaptive immune responses in acute and chronic HCV. In a recent study by Dill and colleagues [29], liver biopsies were performed in 6 patients with acute HCV infection. They found that ISGs were induced in acute infection with IP-10 showing the strongest fold induction of any gene in all 6 patients. Due to the small numbers, they could not comment on the association of ISG expression and response. Interestingly, in the acute patients, the pattern of ISG induction was typical of the response to IFN-γ, rather than to IFN-α, IFN–β or IFN-λ, as is seen in chronic infection. Furthermore, ISG expression levels also correlated with IFN-γ mRNA levels in infiltrating CD8+ T cells suggesting that the adaptive T-cell response may be important. In chronic infection, adaptive immune responses are weak or undetectable and are not augmented with interferon treatment [30]. Rather, viral clearance appears to result from the direct antiviral effects of ISGs induced by interferon. In contrast, in acute HCV infection, there is a robust and broad adaptive response [30]. It is likely that interferon further stimulates the adaptive immune response, which, when combined with the direct antiviral effects of the innate response, is able to control and eradicate infection in the majority of patients [31]. Patients with higher baseline IP-10 levels still had a relatively weak direct antiviral response to interferon and hence had lower rates of RVR, but they were ultimately able to clear infection, likely because of stimulation of the adaptive response by interferon. This is in keeping with the observation that symptomatic acute infection is associated with clearance, a marker of a strong adaptive response targeting the liver [32].

Exposure of hepatocytes to interferon-γ leads to ISG induction but does not result in an interferon-refractory state [29]. In contrast, cells exposed to Type I (α/β) or Type III (λ) interferons are refractory to subsequent treatment with interferon-α [33]–[35]. The levels of ISG induction in chronic HCV infection are strongly correlated with the IL28B genotype, with patients with the treatment-unfavourable (non-CC) genotype showing higher baseline hepatic ISG levels [11]. The mechanism remains unclear but many have speculated and there is some evidence that the IL28B genotype influences the degree of production and/or response to interferon-λ induced by HCV infection [36], [37]. Unlike in the setting of chronic infection, the IL28B genotype was not predictive of early or late treatment responses in this study, however patients with the treatment unfavourable IL28B genotype had higher baseline IP-10 levels. IP-10 levels correlated strongly with HCV RNA suggesting that HCV may be directly driving IP-10 and likely ISG expression. Dill and colleagues may be correct that the predominant interferon produced in response to acute HCV infection is interferon-γ [29], however in patients with very high IP-10 levels, Type I or Type III interferons may also be present, driving higher IP-10 levels and making cells refractory to both endogenous and therapeutic interferon. However, the robust adaptive immune response is still adequate to lead to viral clearance in the majority of patients.

Casrouge and colleagues proposed [27] an alternative explanation to the seemingly paradoxical finding of high ISG activation leading to treatment failure. They showed that IP-10 can undergo cleavage to an inactive version of the chemokine that may act as a dominant-negative by binding the CXCR3 without leading to chemotaxis. Prevention of IP-10 cleavage restored hepatic T-cell infiltration in response to IP-10 in uninfected mice. In our study, only total IP-10 was measured without differentiation of the two isoforms. In chronic infection, Casrouge et al found that circulating IP-10 was predominantly the inactive cleaved form, which should prevent chemotaxis to the liver [27]. The high SVR rate in early HCV infection despite high IP-10 and a poor early response to interferon, would suggest that interferon is still able to promote a strong adaptive response in the liver, which ultimately drives viral clearance. It is possible that high circulating IP-10 levels are more a reflection of ISG preactivation with subsequent poor ISG induction in response to interferon than a marker of poor chemotaxis to the liver. Further studies on cleaved IP-10 will hopefully shed some further light on its relevance in HCV infection.

Ribavirin has been previously shown to affect the second phase decline in HCV RNA during interferon-based therapy [38]. One proposed mechanism for the effect of ribavirin is augmentation of ISG induction by interferon [39], [40]. Previous studies have shown that ribavirin has the greatest effect on the second phase decline in patients with intermediate interferon sensitivity, with minimal benefit seen in those with either very strong or very weak interferon responses [41], [42]. Similarly in this study, ribavirin had little effect in patients with low IP-10 levels at baseline, patients likely to be very responsive to interferon, whereas in those with high baseline IP-10 (>150 pg/mL), ribavirin accentuated the later phase of viral decline, beyond week 4.

This study has some important limitations. Because of the known relationship between IP-10 and treatment outcome in chronic infection, it is possible that the IP-10-RVR relationship seen in this cohort was driven by those with early chronic rather than true acute HCV infection. However IP-10 was associated with RVR after controlling for estimated duration of infection and there was no correlation between IP-10 and SVR as seen in chronic HCV infection. In chronic HCV infection, the relationship between IP-10 and SVR is strongest in patients with genotype 1 infection. The lack of association with IP-10 levels and SVR in this cohort was also seen when the analysis was restricted to those with genotype 1 only, however given the relatively small number of genotype 1 patients (n = 50), it is possible that an association with IP-10 levels and SVR in genotype 1 patients may have been missed. Unfortunately serum was collected prior to recognition of IP-10 cleavage. Post-collection cleavage of IP-10 occurs unless a specific inhibitor is added to the serum at collection and hence it was not possible to accurately determine the ratio of cleaved to uncleaved IP-10 [27]. Finally, although this is one of the larger treatment studies of early HCV infection, the number of patients is still relatively small, limiting the strength of the conclusions.

In summary, baseline IP-10 levels were associated with early viral kinetics but not ultimate treatment outcome to interferon-based therapy in acute or early chronic HCV infection. IP-10 levels correlated with HCV RNA before, during and after treatment. Given previous data showing that patients with high baseline IP-10 are unlikely to spontaneously clear HCV infection they should be prioritized for early interferon-based treatment.
